# Application of the Locking Compression Pediatric Hip Plate™ in children with proximal femoral tumors

**DOI:** 10.1186/s13018-022-03433-6

**Published:** 2022-12-12

**Authors:** Xin Jiang

**Affiliations:** Department of Pediatric Surgery, West China Hospital, Sichuan University, Chengdu, 610041 Sichuan China

**Keywords:** Children, Proximal femur tumor, Locking Compression Pediatric Hip Plate™, Reconstructive surgery, Pathological fracture

## Abstract

**Background:**

Pediatric proximal femoral tumors often present with accumulative and severe bone destruction and are often complicated by pathological fractures and malunion. Such tumors are treated clinically by lesion scraping and graft reconstruction with autologous iliac bone alone or in combination with artificial bone. This study aimed to determine the efficacy of the Locking Compression Pediatric Hip Plate™ in treating pediatric proximal femoral tumors.

**Methods:**

From 2012–2017, the Locking Compression Pediatric Hip Plate™ was applied for internal fixation in 28 children in the Department of Pediatric Surgery. The complications were pathological fractures in 19 patients and multiple lesions in 5 patients. Tumors were removed by tumor curettage and reconstruction with autogenous iliac bone or artificial bone graft. The Locking Compression Pediatric Hip Plate™ was then applied. Postoperative pathological examination confirmed the diagnosis.

**Results:**

The cohort comprised 20 males and 8 females (mean age 7.8 ± 2.9 years). The mean follow-up duration was 26.1 ± 8.1 months (range 18–48 months). Post-treatment radiography showed that the lesions and local pathological fractures were healed in 3.2 ± 0.4 months (range 3–4 months), with no complications. Four patients continued to receive antineoplastic therapy postoperatively. Four patients experienced recurrence in situ, while another four developed distant metastases. The radiographic and joint function findings indicated that the affected limbs had excellent function. The mean Enneking score was 28.7 ± 1.0 points (range 27–30 points).

**Conclusions:**

Internal fixation with the Locking Compression Pediatric Hip Plate™ in children achieves good therapeutic effects. Moreover, the Locking Compression Pediatric Hip Plate™ resolves the shortcomings of external fixation by traditional plaster casts and internal fixation by Kirschner wires and elastic intramedullary screws.

## Background

The proximal femur (including the head and neck, and intertrochanteric and subtrochanteric regions) is one of the most common sites of benign tumors and tumor-like lesions in children. Due to the special anatomical features and biomechanical characteristics of the proximal femur, such lesions readily cause pathological fractures, aseptic necrosis of the femoral head, and malunion of the femoral neck [1]. Diverse clinical methods have been developed to treat proximal femoral tumors; however, given the nature and scope of the lesions, local bone defects, tumor recurrence, malformation, and femoral head necrosis, the surgical treatment of pediatric proximal femoral tumors remains challenging. Furthermore, children are not the epitome of adults. The current discussion focuses on whether operative treatment is necessary and how to reasonably apply internal fixation [2]. The present study aimed to evaluate the effectiveness of the Locking Compression Pediatric Hip Plate™ in treating pediatric proximal femoral tumors.

## Methods

### Patients

This retrospective study included 28 consecutive pediatric patients with proximal femoral tumors treated using the Locking Compression Pediatric Hip Plate™ for internal fixation between February 2012 and February 2017 in the Department of Pediatric Surgery, West China Hospital, Sichuan University, Chengdu, Sichuan. The inclusion criteria were: (1) proximal femoral tumors (whether previously treated or not); (2) age < 15 years. The exclusion criterion was surgery refusal.

The study protocol was approved by the Ethics Committee for Clinical Trials of the West China Hospital (approval no. 2019[797]). The study was registered in the Chinese Clinical Trial Registry (registration number ChiCTR1900026957).

### Operative methods

**Fig. 1:**
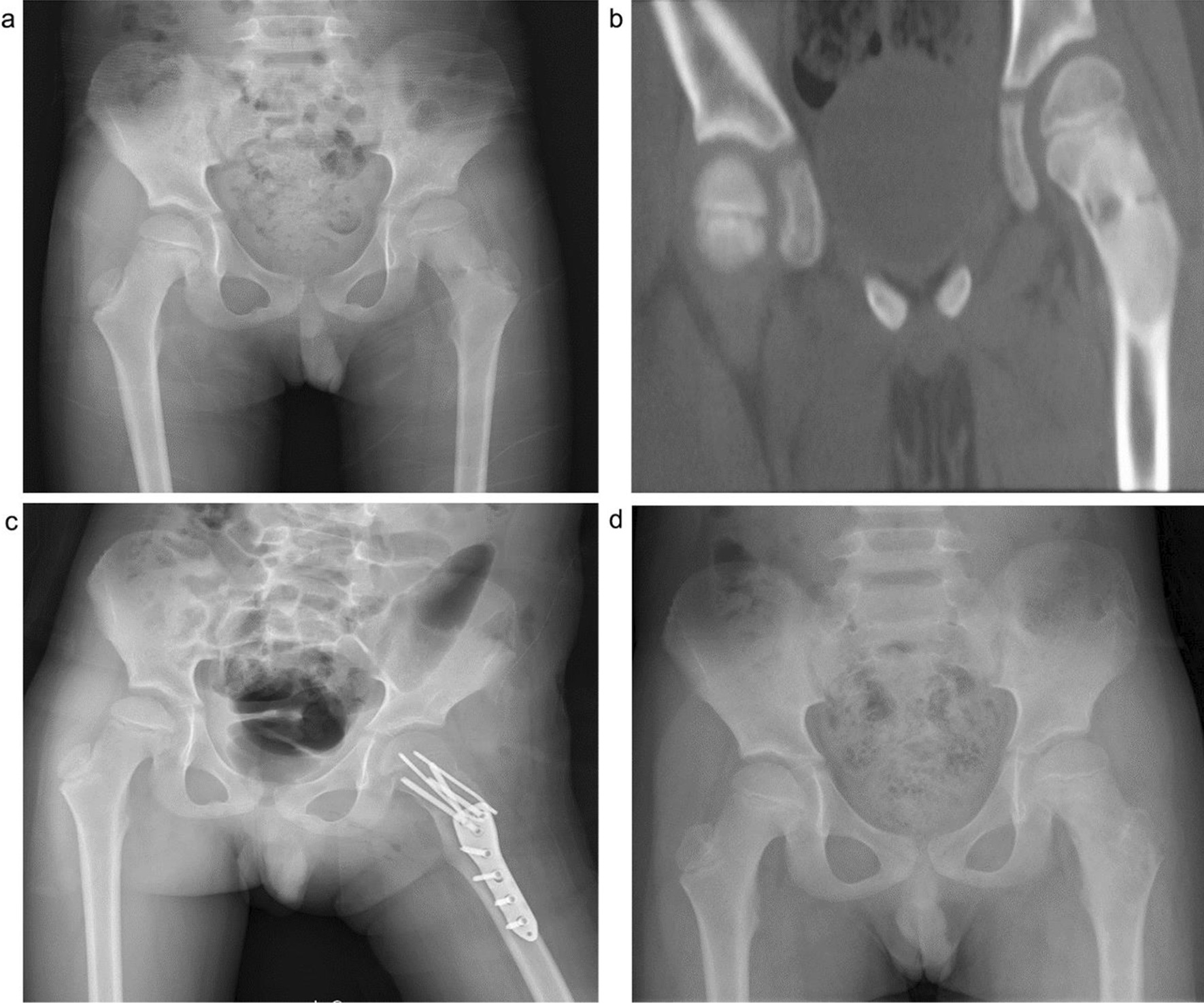
An 8-year-old boy diagnosed with fibrous dysplasia of the left femoral neck with a pathological fracture. Surgical treatment of the left femur tumor was performed by curettage, autogenous iliac bone grafting, and internal fixation with Locking Compression Pediatric Hip Plate™. **a**. Preoperative X-ray; **b**. Preoperative CT; **c**. Postoperative; and **d**. Two years after operation

**Fig. 2:**
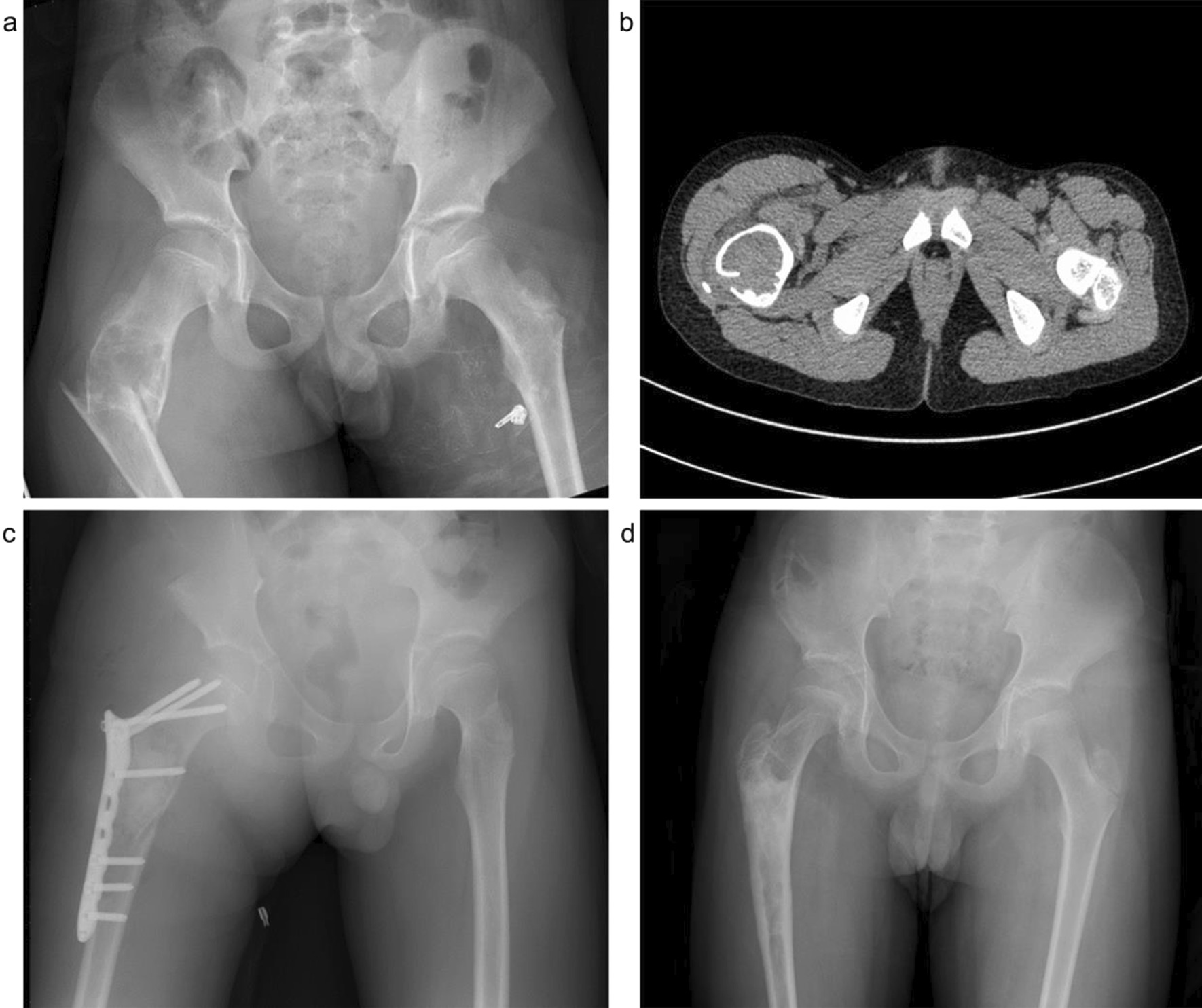
A 12-year-old boy diagnosed with simple bone cyst of right femoral trochanter with a pathological fracture. Surgical treatment of the right femur tumor was performed by curettage, artificial bone + autogenous iliac bone grafting; the pathological fracture was treated by orthopedic reduction and internal fixation with Locking Compression Pediatric Hip Plate™. **a**. Preoperative X-ray; **b**. Preoperative CT; **c**. Postoperative; and **d**. Two years after operation

The operation plan was determined based on radiography, CT, and radioisotope (99Tcm) bone scanning. The horizontal position was adopted for surgery. The affected side was bolstered up, and a lateral femoral incision was performed with the greater trochanter as the marker to fully expose the lesion site. The tumor tissue was completely scraped under direct vision and sent to the pathology laboratory for definitive diagnosis; care was taken not to damage the normal epiphyseal plate. Electrocautery was then performed to burn the residual cavity walls. Subsequently, an alcohol-soaked gauze was used to fill the residual cavity for 5 min, followed by rinsing of the residual cavity. As the bone tumors had severely damaged the normal bone structure, the Locking Compression Pediatric Hip Plate™ was used for internal fixation as primary treatment to prevent pathological fractures or treat existing fractures. Five patients with concomitant pathological hip varus deformity were simultaneously treated with internal fixation to maintain a normal neck shaft angle. Finally, the defects were reconstructed with bone grafts. Eight patients were treated with autologous iliac bone grafts, while 20 patients with large bone defects were treated with autologous iliac/artificial bone grafts. The β-TCP artificial bone was used for bone filling, and the mass of the artificial bone grafts ranged from 5 to 15 g (Figs. 1, 2). The decision as to whether to continue antitumor treatment postoperatively was based on the pathological examination results.

### Postoperative management

The affected limb was externally fixed with herringbone plaster, and patients were not allowed to weight-bear on the affected limb for 2–3 months postoperatively. Cephalosporin was administered within 24 h to prevent infection. At 2 weeks postoperatively, the wound sutures were removed and the hip herringbone plaster was divided into upper and lower parts and opened. Patients were then assisted to sit up and start moving their hips and knees without weight-bearing. A postoperative radiographic examination was performed, and limb function exercises were started when possible in accordance with the nature of the tumor and the healing of the defect and any fractures. One patient had neuroblastoma with multiple metastatic bone lesions throughout the whole body complicated by pathological fractures postoperatively; this patient was transferred to the pediatric oncology department for antitumor treatment.

The timing of hardware removal depended on the healing time of the bone defects and pathological fractures, and whether the lesions recurred after surgery. As the patients were children, the hardware was removed as soon as possible to avoid complications. Most patients had the hardware removed about 2 years after surgery. If epiphyseal injury occurred, it was recommended to remove the hardware as early as possible (e.g., at 6 months postoperatively).

## Results

### Patient baseline characteristics

The 28 patients in the study population accounted for 58.3% of all children with proximal femoral tumors (48 patients) treated in the same period. The study cohort comprised 20 males and 8 females with an average age of 7.8 ± 2.9 years (range 2–14 years). There were 6 patients with simple bone cysts, five with aneurysmal bone cysts, seven with eosinophilic granulomas, eight with fibrous dysplasia, one with neuroblastoma, and one with McCune–Albright syndrome. The average disease course was 2 months (range 1 day to 12 months). The complications comprised pathological fractures in 19 patients and multiple lesions throughout the body in five. Five patients presented with chronic claudication and gait abnormalities of the lower limbs, nine had claudication with intermittent hip and thigh pain, and 14 were asymptomatic but had lesions detected during examination at the time of admission for limb swelling, pain, and movement limitation after trauma. Seven patients were diagnosed with tumor lesions before admission and had been treated by immobilization and observation, but were admitted to the emergency department due to pathological fractures caused by trauma or other reasons. After admission, all patients underwent routine radiography and CT examinations, which showed that the bone tumors were located in the proximal femur, including the head and neck (13 patients), intertrochanteric region (8 patients), and subtrochanteric region (7 patients). Most patients had pronounced bone destruction, especially those with pathological fractures involving two regions. A total of 20 patients underwent radioisotope (99Tcm) whole-body bone scans, which revealed multiple bone lesions in five patients (Table 1).

**Table 1:** Patient characteristics

Case	Age/gender	Diagnosis	Location of the tumor				Pathological fracture	Multiple tumors	Daily symptoms		
			Head	Neck	Trochanteric	Subtrochanteric			Limp	Pain	Normal
1	8/M	FD		√	√		√		√		
2	12/M	SBC			√	√	√				√
3	10/M	OB	√	√	√		√		√	√	
4	14/M	FD			√	√					√
5	3/F	LCH	√	√			√	√	√	√	
6	4/M	MAS		√	√	√	√	√	√	√	
7	9/M	ABC		√	√	√	√				√
8	9/M	SBC			√		√				√
9	6/F	SBC		√	√		√				√
10	5/M	LCH		√	√				√		
11	13/M	FD	√	√	√		√	√	√	√	
12	12/M	ABC		√	√	√			√	√	
13	7/M	SBC		√	√		√				√
14	8/M	SBC		√	√	√	√				√
15	8/M	FD	√	√	√		√	√	√	√	
16	9/F	LCH		√					√		
17	4/M	LCH			√	√					√
18	10/M	FD	√	√							√
19	6/M	ABC		√	√	√	√		√		
20	9/F	LCH		√	√						√
21	2/F	NB		√	√	√	√	√	√	√	
22	7/M	ABC		√			√				√
23	10/F	LCH		√	√	√	√				√
24	9/M	FD	√	√	√						√
25	7/F	FD		√	√				√	√	
26	6/M	SBC		√	√		√				√
27	4/F	FD			√	√	√		√		
28	8/M	LCH		√	√		√		√	√	

*F*, Female; *M*, Male; *SBC*, Simple bone cyst; *ABC*, Aneurysmal bone cyst; *LCH*, Langerhans cell histiocytosis; *FD*, Fibrous dysplasia; *OB*, Osteoblastoma; *NB*, Neuroblastoma; *MAS*, McCune–Albright syndrome

### Clinical outcomes

The average follow-up duration was 26.1 ± 8.1 months (range 1–4 years). Radiography showed that the lesions and local pathological fractures were healed after treatment, with an average healing time of 3.2 ± 0.4 months (range 3–4 months). There were no complications, including nail breakage, nail detachment, plate loosening, broken plate, hip varus, or joint movement disorder. Four patients continued antineoplastic therapy postoperatively. Four patients had recurrence in situ (two underwent reoperation and two received continuous observation), and four showed distant metastases (two underwent reoperation and two received continuous observation). Repeat radiography and comprehensive evaluation of joint function using the evaluation criteria for therapeutic effects proposed by Mankin et al. [3] showed that all affected limbs had excellent function after treatment. The average function score based on the Enneking system [4] was 28.7 ± 1.0 points (range 27–30 points) (Table 2).

**Table 2:** Clinical outcomes in the 28 patients

Case	Bone grafting		Bed-rest time (month)	Healing time (month)	Postoperative tumor therapy	Recurrence in situ	Distant metastasis	Follow-up time (month)	Enneking score
	Autogenous iliac bone	Autogenous iliac bone + Artificial bone							
1	√		2	3				24	30
2		√	4	3.5				24	29
3		√	3	4				36	29
4		√	3	3				24	30
5		√	3	3	√		√	48	30
6	√		3	3	√	√	√	48	27
8		√	3	3		√		18	29
9		√	3	3				36	30
10	√		2	3				24	28
11		√	3	4			√	24	27
12		√	3	3				18	28
13		√	3	3				24	30
14		√	3	3				18	30
16		√	3	3				36	28
17	√		3	4				24	28
19		√	3	3		√		24	28
20		√	3	4				24	28
21	√		3	3	√		√	24	28
22		√	3	3				18	28
23		√	3	3.5	√			24	29
24		√	3	4		√		24	28
25	√		3	3				24	29
26		√	2	3				24	30
27	√		2	3				24	29
28		√	3	3				18	28

## Discussion

In the present study, internal fixation with the Locking Compression Pediatric Hip Plate™ achieved a good therapeutic effect in pediatric patients with proximal femoral tumors.

According to the 4th edition of the WHO classification of bone tumors published in 2013, the simple bone cyst, aneurysmal bone cyst, Langerhans cell histiocytosis, and fibrous dysplasia are considered “tumors of undefined neoplastic nature” [5]. For these types of tumors, most scholars still advocate long-term clinical observation after pathological examination and diagnosis of isolated lesions, and appropriate local conservative measures such as symptomatic treatment [6–8]. However, due to the special anatomical and biomechanical characteristics of the proximal femur, such lesions readily cause pathological fractures, aseptic necrosis of the femoral head, and malunion of the femoral neck [9]. Furthermore, compared with adults, children have a poor sense of safety and experience more severe tumor destruction and multiple complications. Therefore, we proposed that for pediatric patients with pathologic fractures and those with a high risk of pathological fractures (i.e., those who have not yet developed pathological fractures but have an unclear diagnosis or significantly decreased anatomical strength), operative treatment should be initiated with the main goals of removing the lesions, restoring bone strength, and maintaining normal limb function to the maximum extent.

Due to the particularities of the physiology and anatomical structure of children, multiple surgical methods have been applied in clinical practice for proximal femoral lesions, including lesion scraping, residual cavity treatment, bone defect repair, internal fixation, and artificial prosthesis replacement [10–12]. In the present study, under the condition of radical tumor resection, the normal bone and soft tissue surrounding the lesions were protected to achieve a low recurrence rate while maintaining joint function to the maximum extent. We performed lesion scraping and graft reconstruction with autologous iliac bone (or autologous bone combined with artificial bone) in children with proximal femoral tumors, and long-term follow-up indicated a good healing effect, corroborating the findings of a previous study [13].

**Fig. 3:**
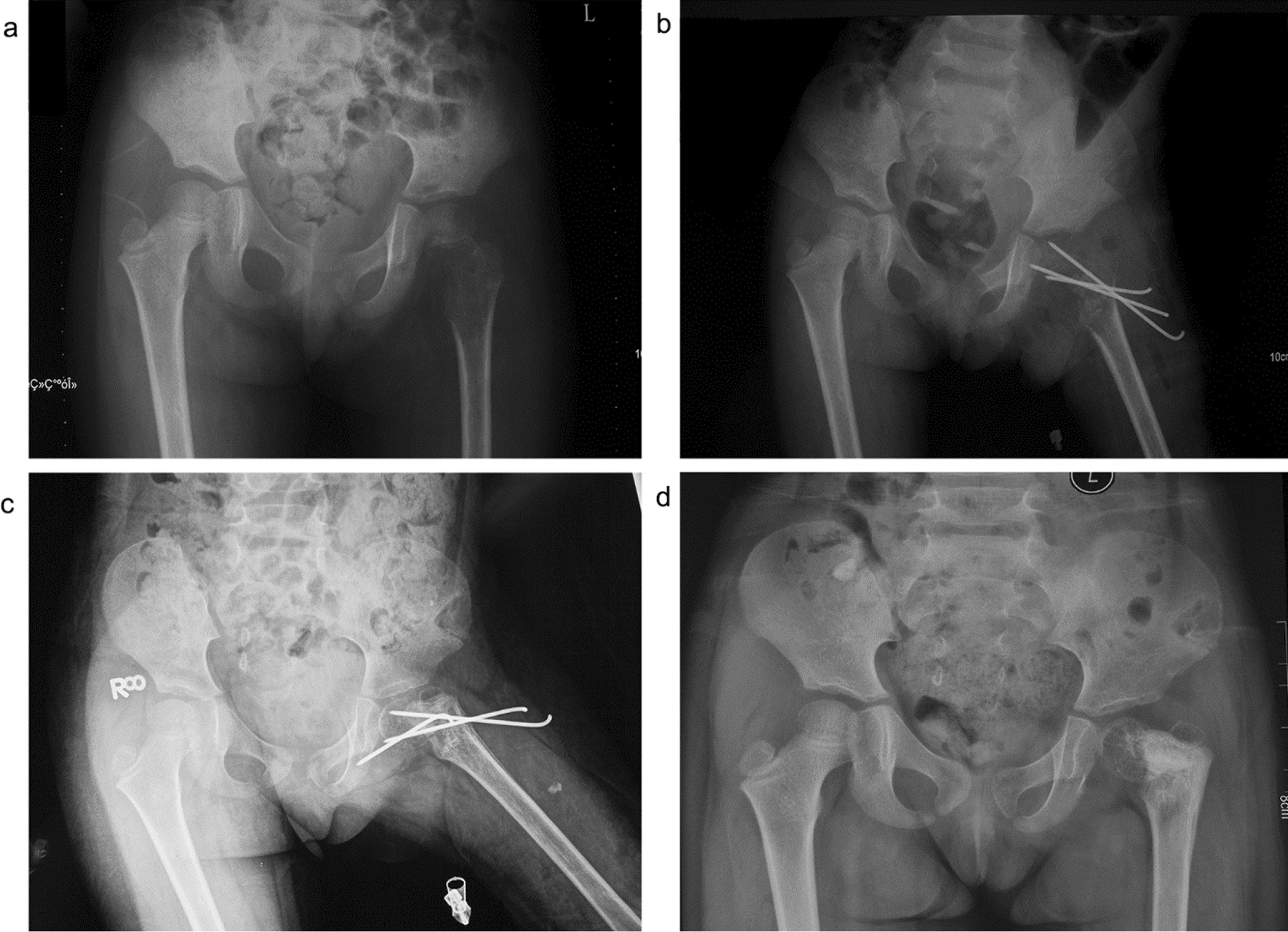
A 5-year-old girl diagnosed with eosinophilic granuloma of the left femoral neck. Surgical treatment of the left femur tumor was performed by curettage, and Kirschner’s needle internal fixation. **a**. Preoperative; **b**. Postoperative; **c**. Kirschner wire loosening and displacement 3 months after operation; and **d**. Two years after operation, coxa varus

**Fig. 4:**
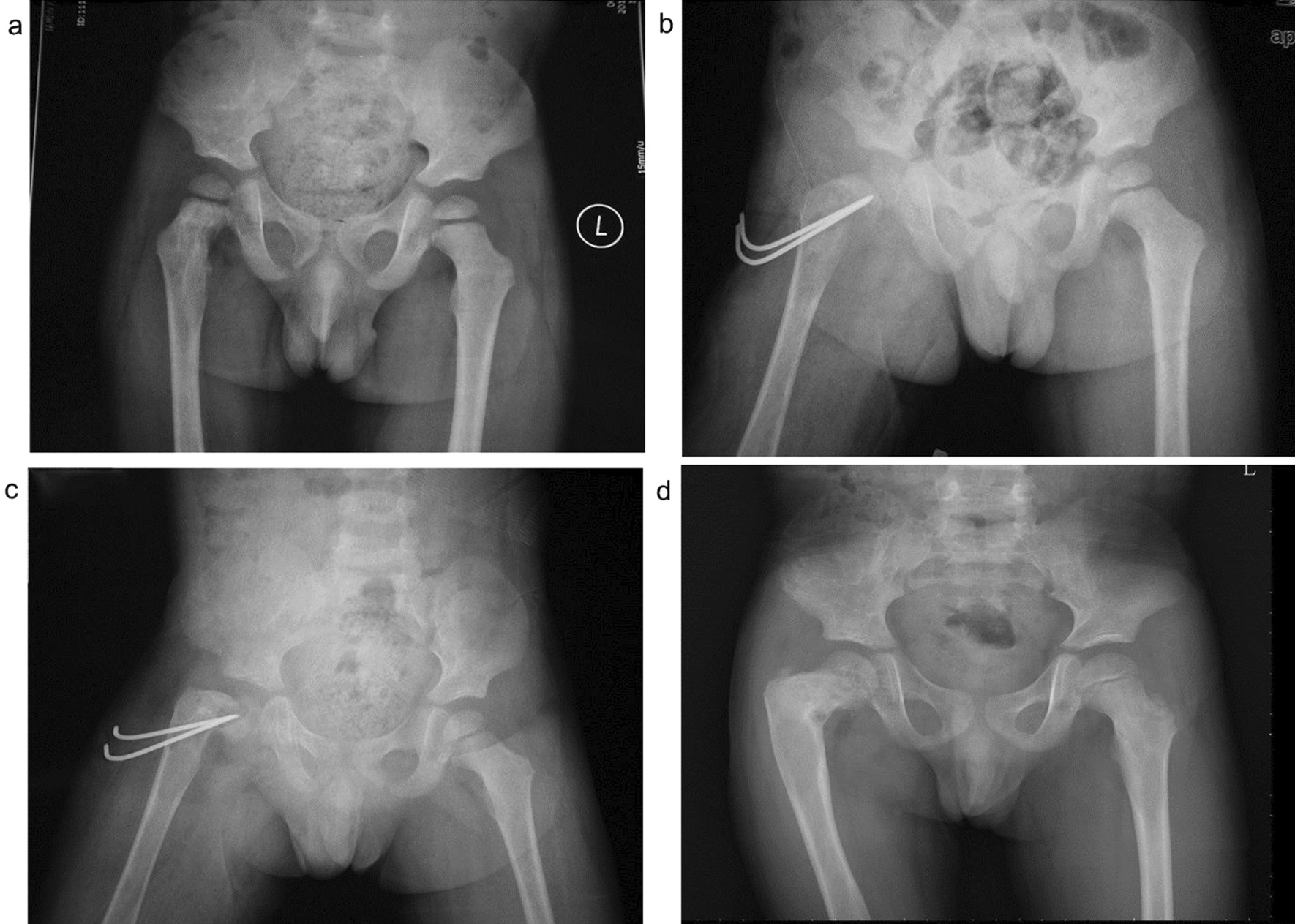
A 2-year-old boy diagnosed with fibrous dysplasia of the right femoral neck. Surgical treatment of the right femur tumor was performed by curettage, and internal fixation with a Kirschner’s needle. **a**. Preoperative; **b**. Postoperative; **c**. 3 months after operation; and **d**. Two years after operation, coxa varus

**Fig. 5:**
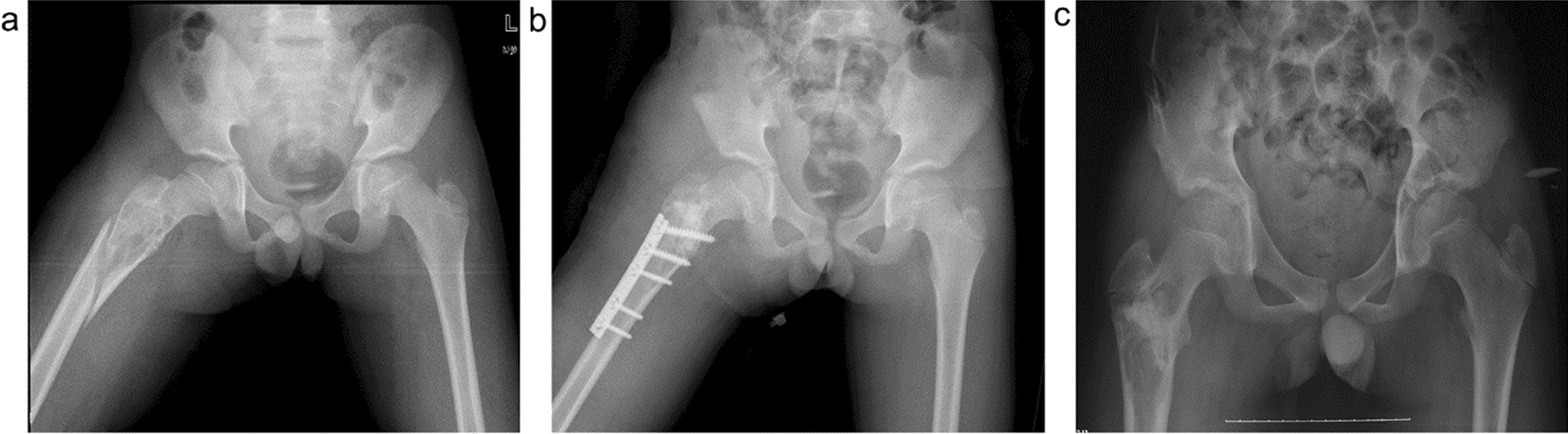
A 10-year-old boy diagnosed with a right proximal femur cyst and a pathological comminuted fracture. Surgical treatment of the right femur tumor was performed by curettage, with artificial bone + autogenous iliac bone grafting. The pathological fracture was treated by orthopedic reduction and internal fixation with a straight steel plate. **a**. Preoperative; **b**. Postoperative; and **c**. Two years after operation

For patients with limited lesions, non-significant decreases in anatomical structure stability, or an unclear pathological diagnosis, there is no indication for internal fixation during primary treatment. However, for patients with fractures or severe bone damage and a high risk of pathological fracture (i.e., no pathological fracture has occurred, but the diagnosis was unclear and the anatomical strength was significantly reduced, for example, the bone defect is 1/3 of the femoral diameter), the majority of scholars advocate internal fixation to minimize complications such as femoral head and neck deformity and achieve early joint mobilization, which could improve the quality of life and facilitate care [14]. According to the tumor lesion sites and the degree of bone destruction, appropriate internal fixators are selected, including Kirschner wires, elastic intramedullary nails, hollow screws, straight plates, angle plates, dynamic hip screw systems, and hip locking compression plates, each of which has specific advantages and disadvantages [15–18]. Kirschner wires and intramedullary nails are considered to cause limited damage to anatomical structures and are commonly applied [19–21]. However, inadequate internal fixation strength is likely to result in internal fixator loosening or deformation, or even failure of hardware removal due to internal fixator rupture. Furthermore, local malunion of the lesion may occur, resulting in hip varus or ischemic necrosis of the femoral head (Figs. 3, 4, 5). It is important that any internal fixation does not damage the epiphysis and maintains the anatomical structure stability to the maximum extent to prevent complications such as ischemic necrosis of the femoral head and malunion of the femoral head and neck.

**Fig. 6:**
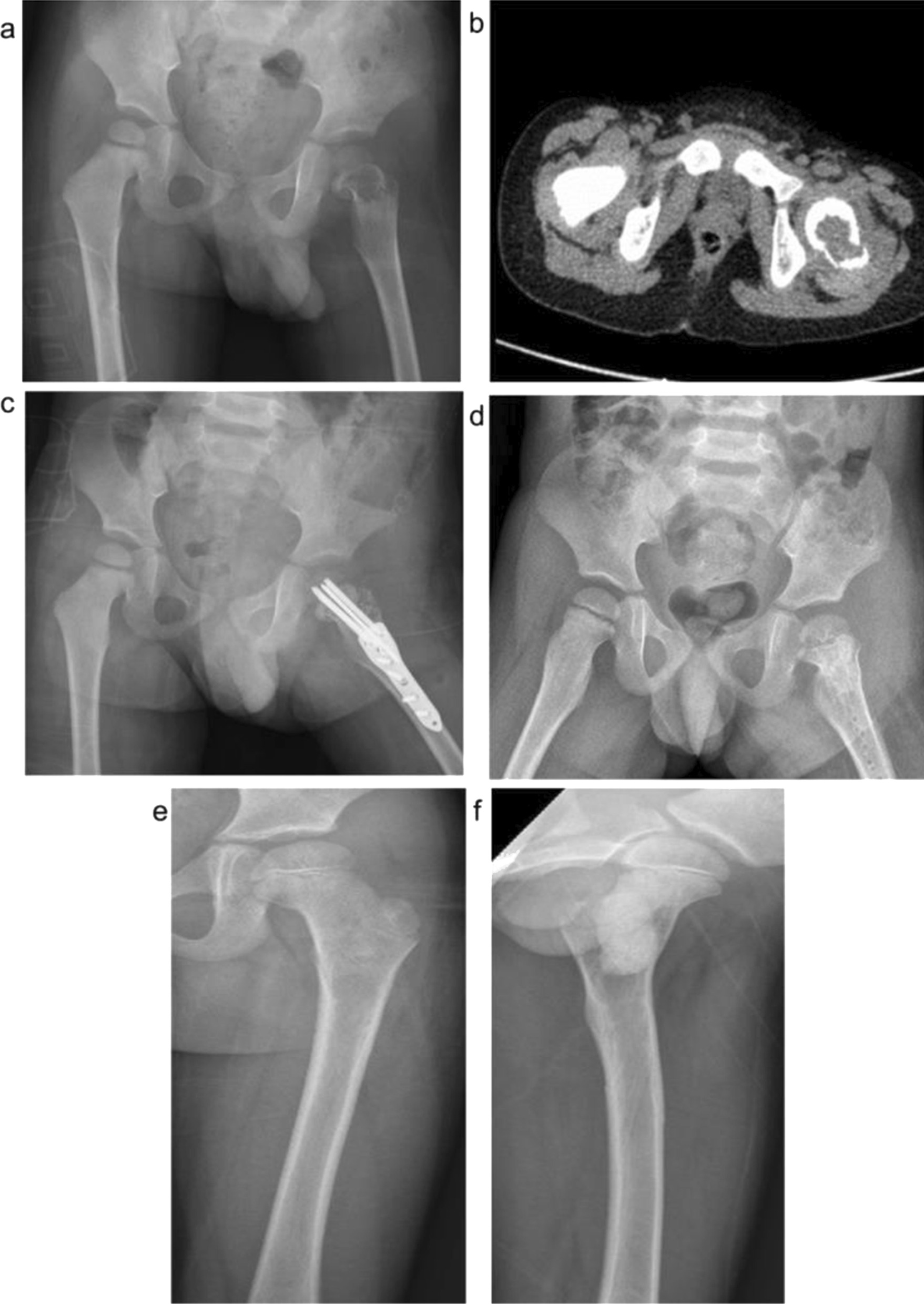
A 3-year-old girl diagnosed with Langerhans cell histiocytosis of the left femoral head and neck with a pathological fracture. Surgical treatment of the left femur tumor was performed by curettage and artificial bone + autogenous iliac bone grafting. The pathological fracture was treated by orthopedic reduction and internal fixation with the Locking Compression Pediatric Hip Plate™. **a**. Preoperative X-ray; **b**. Preoperative CT; **c**. Postoperative; **d**. 6 months after operation; **e**. 4 years after operation, positive X-ray; and **f**. 4 years after operation, lateral X-ray

In the present study, the Locking Compression Pediatric Hip Plate™ was used to treat pediatric proximal femoral tumors. The locking plate with a stable angle is the first choice recommended by the AO for the treatment of subtrochanteric femoral fractures in children and is widely used in the treatment of pediatric hip dislocation, hip varus, and proximal femoral fracture [22, 23]. The advantages of this technique are that: (1) the screw self-locks with the bone plate to form an integration, which favors the healing of the broken end; (2) pressurized contact is not required between the bone plate and surface; (3) the locking state confers good angular stability and fixation strength, with no possible screw swing in the hole; (4) angular fixation greatly reduces bone loss after reduction of corrective osteotomy intra- and postoperatively [24, 25]. However, the Locking Compression Pediatric Hip Plate™ also has certain shortcomings in children. For example, “minimally invasive” surgery is not possible, and a second operation is required to remove the internal fixator. In addition, there is a risk of secondary injury arising from penetration through the epiphyseal plate (Fig. 6).

Due to the presence of large bone defects, 8 patients were treated with autologous iliac bone grafts, and 20 were treated with autologous iliac/artificial bone grafts. In clinical practice, it is very difficult to deal with the bone defects resulting from the removal of bone tumors. To maintain the integrity of the bone structure and the stability of the bone and adjacent joint functions, the commonly used repair methods include autologous bone, allograft bone, tissue-engineered bone, and artificial bone grafting. However, these methods each have disadvantages. The source of autologous iliac bone is limited, and bone removal surgery increases the trauma and the risk of complications. Allogeneic bone has the disadvantages of immune rejection, cross-infection, and slow healing. There is no report on the application of tissue-engineered bone in children. Although phosphate-containing artificial bone has good biocompatibility, it has a relatively slow healing time and absorption. For children with large bone defects, autogenous iliac bone transplantation is still the preferred bone transplantation strategy. For severe bone defects, such as those weighing more than 15–20 g, we used autologous iliac bone and artificial bone mixed with a bone graft. Long-term follow-up showed that the bone defects had healed well.

The present study has some limitations. First, this was a retrospective study performed in a single center with a limited number of patients. Second, there is the possibility of selection bias, and the statistical power may be too low to allow our findings to be generalized. Third, there was no control group (e.g., pediatric patients treated using another currently used method). Therefore, further well-designed large sample studies are warranted to confirm the present results.

## Conclusions

Internal fixation with the Locking Compression Pediatric Hip Plate™ is an effective treatment method for proximal femoral lesions in children. This technique mitigates the inadequacy of external fixation by traditional plaster casts and internal fixation by Kirschner wires and elastic intramedullary screws. Moreover, postoperative functional exercise can be performed at an early stage to reduce joint dysfunction and promote tumor healing, leading to good therapeutic effects.

## Data Availability

The datasets used and/or analyzed during the current study are available from the corresponding author on reasonable request.

## Abbreviations

LCP: Locking Compression Plate
AO: Arbeitsgemeinschaft für Osteosynthese
